# Examining the impact of inducible ischemia on myocardial fibrosis and exercise capacity in hypertrophic cardiomyopathy

**DOI:** 10.1038/s41598-020-71394-z

**Published:** 2020-09-29

**Authors:** Maan Malahfji, Alpana Senapati, Dany Debs, Clara Angulo, Yang Zhan, Sherif F. Nagueh, Dipan J. Shah

**Affiliations:** 1 Department of Cardiology, Houston Methodist DeBakey Heart and Vascular Center, Houston, TX USA; 2 Division of Cardiology, Departement of Internal Medicine, University of Saskatchewan, Saskatoon, Canada

**Keywords:** Cardiology, Cardiac hypertrophy

## Abstract

Myocardial ischemia in hypertrophic cardiomyopathy (HCM) is associated with poor outcomes. Vasodilator stress cardiac magnetic resonance (CMR) can detect and quantitate inducible ischemia in HCM patients. We hypothesized that myocardial ischemia assessed by CMR is associated with myocardial fibrosis and reduced exercise capacity in HCM. In 105 consecutive HCM patients, we performed quantitative assessment of left ventricular volume and mass, wall thickness, segmental wall thickening percent, segmental late Gadolinium enhancement (LGE), and extracellular volume fraction (ECV). Time-signal intensity curves of first pass perfusion sequences were generated for each segment at stress and rest. A myocardial perfusion reserve index (MPRI) (stress/rest slope) was calculated. Patients who underwent an echocardiographic (n = 73) and cardiopulmonary exercise test (n = 37) within 30 days were included. The mean age was 53.2 ± 15.4 years; 60% were male, and 82 patients had asymmetric hypertrophy. Segments with end diastolic thickness ≥ 1.2 cm had a higher burden of LGE (4.1% vs 0.5% per segment), reduced MPRI (2.6 ± 1.5 vs 3.1 ± 1.8) and reduced thickening percent (48.9 ± 41.7% vs. 105.3 ± 59.5%), (P < 0.0001 for all comparisons). Patients with ischemia (any segment with MPRI < 2) were more likely to have dynamic left ventricular outflow tract (LVOT) obstruction (63.3% vs 36.7%, P = 0.01), to be smokers (17% vs 6.9%, P = 0.04), and had a higher ECV (30% vs 28%, P = 0.04). The total LGE burden was similar between the two groups (P = 0.47). Increasing ischemia burden (number of segments with MPRI < 2) was associated with worsened ventilatory efficiency (VE/VCO2) (P < 0.001) but not peak oxygen consumption or anerobic threshold (P > 0.2). In a patient-level multivariable logistic regression model, only LVOT obstruction remained a significant predictor of ischemia burden (P = 0.03). Myocardial ischemia by CMR is associated with myocardial segmental dysfunction and interstitial fibrosis, as assessed by ECV, in HCM patients, even in segments free of LGE. Conversely, quantitative ischemia burden was not associated with replacement fibrosis as assessed by total LGE burden. Patients with ischemia had greater prevalence of dynamic LVOT obstruction; and in a subset of patients with cardiopulmonary exercise testing, ischemia burden was associated with worsened ventilatory efficiency.

## Introduction

Hypertrophic cardiomyopathy (HCM) is the most common heritable cardiomyopathy, with a prevalence recently estimated as high as 1 in 200 live births^1^. The natural history of HCM varies from a benign asymptomatic course to symptoms of chest pain or dyspnea, and in advanced stages to heart failure and sudden death^2^. Myocardial ischemia is an important pathophysiologic process in HCM and is associated with left ventricular adverse remodeling and systolic dysfunction^3,4^. Potential mechanisms include increased oxygen requirements due to hypertrophy, diastolic dysfunction and elevated filling pressures, abnormalities of intramyocardial arterioles, abnormal coronary flow dynamics in part due to left ventricular outflow tract (LVOT) obstruction^5–7^.

Cardiovascular magnetic resonance (CMR) is an important imaging modality in diagnosing and risk stratifying HCM patients^8,9^. It is a validated non-invasive method for quantitation of myocardial replacement fibrosis using the late gadolinium enhancement (LGE) technique^10^, as well as myocardial interstitial fibrosis using extracellular volume fraction (ECV)^11,12^. Vasodilator stress CMR allows for detection and quantitation of myocardial ischemia, and the degree of ischemia assessed by CMR has recently been linked to progression of LGE^13^. Assessment of myocardial ischemia, however, is not part of routine clinical assessment of HCM patients by CMR, particularly those considered to be low-intermediate risk for SCD^14,15^. We hypothesized that myocardial ischemia assessed by vasodilator stress CMR will be associated with myocardial fibrosis and reduced exercise capacity. Our second objective was to assess the predictors of ischemia on CMR.

## Methods

**Figure 1:**
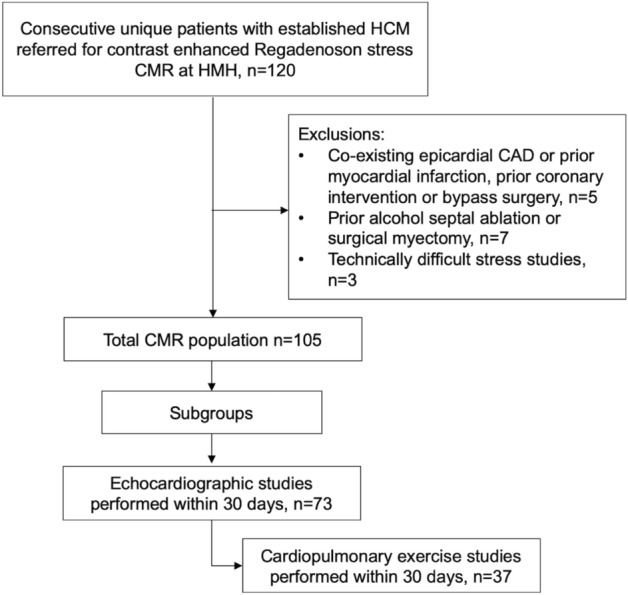
Flow chart shows the exclusion criteria and subgroups. HCM, Hypertrophic cardiomyopathy; HMH, Houston Methodist Hospital; CMR, Cardiac magnetic resonance; CAD, Coronary artery disease.

The study was approved by the institutional review board at Houston Methodist Hospital and informed written consent obtained from all patients. All studies were performed in accordance with relevant guidelines and regulations. We included 105 consecutive adult patients from the DEBAKEY CMR Registry (NCT04281823), a prospective institutional registry. The studies were performed between July 2011 and June 2017. We included patients > 18 years old confirmed to have HCM according to guidelines^8^. We excluded patients who had prior septal reduction therapy, epicardial coronary artery disease (CAD), prior myocardial infarction, late gadolinium enhancement (LGE) in a CAD pattern, implanted cardiac devices, claustrophobia, or glomerular filtration rate < 30 mL/min per 1.76 m^2^. Figure 1 shows an overview of the study and exclusion criteria.

### CMR imaging protocol

Patients were asked to refrain from caffeine for 24 h before Regadenoson stress CMR. Most procedures were completed in approximately 45 min. Studies were performed on 3 T and 1.5 T CMR systems (Siemens Avanto, Aera, Verio, and Skyra, Siemens, Erlangen, Germany). Steady state free precession sequences were used to acquire a series of short axis cine images and standard long axis views (2, 3, 4 chamber) during single breath holds, using retrospective ECG gating with slice thickness of 6, 4 mm gap, echo time = 1.33 ms, temporal resolution per phase 35–40 ms, in-plane resolution ~ 1.5–2.0 × 1.5–2.0 mm/pixel, and flip angle of 45–75 degrees. The ECG triggered modified Look Locker inversion recovery (MOLLI) sequence was employed for assessment of myocardial T1 relaxation times in a mid-ventricular slice. Native myocardial T1 relaxation time was measured before administration of contrast^16^. Regadenoson 0.4 mg (Astellas, Northbrook, Illinois, USA) was injected over approximately 10 s into a peripheral vein followed by a 5 mL saline flush. The stress perfusion sequence was started within 30–60 s of Regadenoson injection. Gadolinium-based contrast (0.15 mmol/kg gadopentetate dimeglumine or gadoterate meglumine) was infused at 3.5–4.5 ml/s followed by a saline flush (50 ml) via an antecubital vein (dose split equally for stress and rest perfusion). Patients routinely received aminophylline 100 mg intravenously for reversal of hyperemia after stress images were acquired. Rest perfusion was performed ~ 10 min after reversal.

LGE imaging was performed ~ 10 min after intravenous injection of the second dose of gadolinium contrast (rest perfusion imaging) using an ECG gated inversion recovery gradient echo sequence in identical locations to the cine images. Scanning parameters included slice thickness of 6 mm, gap 4 mm, voxel size 2.1 × 1.6 × 6 mm, TE = 1.15, and flip angle = 20°^16^. Acquisition scans for post contrast T1 mapping were performed after the standard delayed enhancement acquisition protocol for a mid-ventricular matching slice, approximately 15–20 min after the infusion of the contrast agent. Parameters for MOLLI technique include slice thickness of 6 mm, voxel size 2.1 × 1.6 × 6 mm, TE = 1.09, TR = 675, flip angle = 35°, twofold parallel imaging. Precontrast 5(3)3 and post-contrast 4(1)3(1)2 heart beat sampling schemes were used on the 1.5-T scanner and pre-contrast 5(4)2 and post-contrast 4(1)2(2)2 heart beat sampling schemes on the 3.0-T scanner. Shimming and delta frequency adjustments were applied to minimize off-resonance artifacts^16^.

### CMR imaging analysis

HCM phenotype was assessed by identifying the predominant location of hypertrophy (asymmetric septal hypertrophy, concentric hypertrophy, apical hypertrophy). One experienced observer performed quantification of left and right ventricular ejection fraction and volumes on a series of short axis slices from the base to the apex of each ventricle. The endocardial borders were manually traced for end diastolic and end systolic images for all slices. Quantitative analysis of segmental thickness and segmental systolic function (thickening percent) was recorded according to the American Heart Association’s 16 segment model. Papillary muscles were excluded from ventricular volumes. LV mass was calculated by tracing LV epicardial and endocardial borders at end diastole and assuming myocardial density of 1.05 g/ml. CMR42 Version 5.6 (Circle Cardiovascular, Calgary, Canada) was used for all analyses.

### First pass perfusion analysis

**Figure 2:**
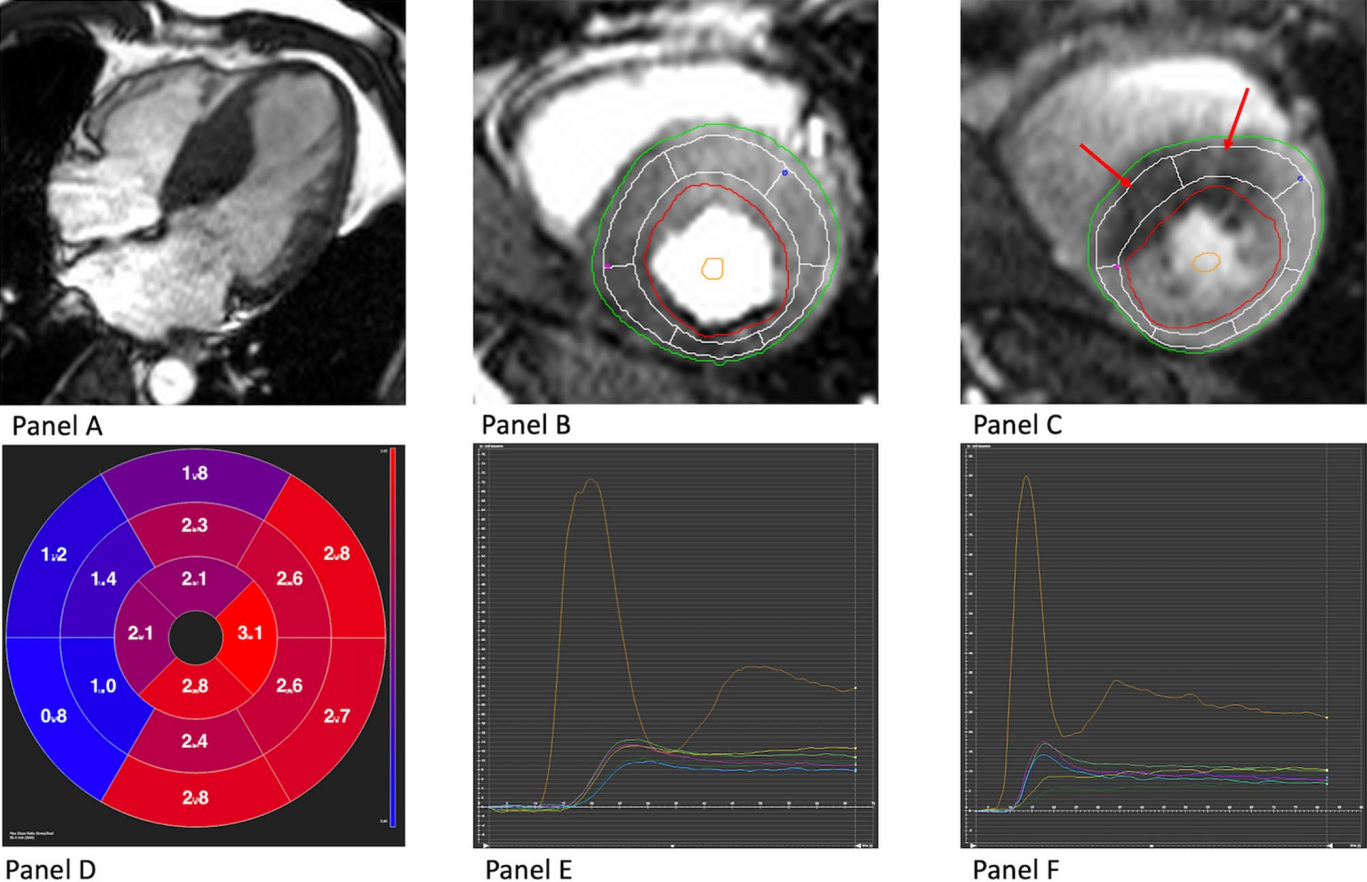
Semiquantitative analysis of MPRI. A case example of a 58 years old patient with hypertrophic cardiomyopathy and septal thickness of 2.7 cm, panel **A**. Panels **B** and **C** show derivation of MPRI at rest and stress respectively. Red arrows highlight segments with reduced flow at stress. Panels **E** and **F** show enhancement curves of myocardial segments at rest and stress respectively, with the arterial input function in orange. Panel **D** shows a polar plot with reduced MPRI measures in the septum. MPRI, myocardial perfusion reserve index; LVOT, left ventricular outflow tract. Patient consent for publishing anonymized figure was obtained.

In semiquantitative perfusion analysis, time-signal intensity curves of motion corrected perfusion sequences were generated for each myocardial segment using semiautomated tracking of the endocardial and epicardial borders with manual correction to exclude artifacts. Corresponding time signal intensity curves were derived for each myocardial segment, and the maximal slope of these curves was reported for each of the 16 myocardial segments. This analysis was repeated for rest perfusion imaging and a myocardial perfusion reserve index (MPRI) (stress/rest slope) calculated for each segment. Baseline correction was applied on the rest perfusion sequences to account for remaining gadolinium contrast signal from the stress sequences performed first. An MPRI below 2 was considered abnormal^17^. Figure 2 shows an example case.

### Late Gadolinium enhancement analysis

Manual endocardial and epicardial contour tracing was performed for all short-axis segmented LGE images and the full width half maximum technique signal threshold was used in LGE quantitation. Total LGE mass was expressed as a percentage of the LV. Percent LGE in each segment was also calculated similar to perfusion slopes and percent thickening.

### T1 mapping analysis

Analysis of T1 values was performed using manually contoured regions of interest, for both pre and post contrast midventricular slices^16^. Extracellular volume (ECV) was calculated as: ECV = ∆R1 myocardium/∆R1 blood × (100 − HCT), where ∆R1 myocardium or change in relaxation rate is given by: 1/T1 myocardium post contrast injection—1/T1 myocardium pre-contrast injection, and ΔR1 blood = 1/T1 blood-post contrast—1/T1 blood-pre-contrast administration. HCT refers to hematocrit obtained at the time of CMR. By this technique an estimate of T1 is encoded in the intensity of each pixel. ECV, T1 pre-contrast and T1 post contrast values were calculated separately for each of the six segments in the midventricular slice before and after contrast administration. We applied the ECV analysis to the six segments at the mid-ventricular level similar to other investigators^18^. Measurements were calculated as an average of all 6 segments. An offset of 20% from epicardial and endocardial contours was applied in the perfusion and T1 sequences to reduce bias from partial volume effects.

Each CMR imaging technique was blindly scored on separate days without knowledge of clinical data or echocardiographic findings, which were collected by other observers. Interobserver variability was assessed in 10 random patients by a blinded experienced CMR reader. First pass perfusion measurements, LGE, and ECV measures had excellent correlation (Interclass correlation coefficient = 0.94, 0.94, 0.96, respectively, P < 0.001).

### Echocardiographic and cardiopulmonary exercise

Patients who underwent an echocardiographic study within 30 days of the CMR study were included (n = 73). Echocardiographic measurements were performed by an independent observer without knowledge of CMR findings. The remainder of patients were referrals from other institutions thus echocardiographic studies were not available for analysis. Dynamic LVOT obstruction was quantified at rest, with the Valsalva maneuver, and with exercise if indicated (six patients had exercise echocardiography, and two of them had no LVOT obstruction at rest but provokable LVOT obstruction with exercise echocardiography)^16^. In patients who did not have an echocardiographic study available for analysis within 30 days of CMR, we used CMR findings or available echocardiographic studies outside of the 30 day window, to determine the presence of LVOT obstruction. This was done only for the purposes of ESC risk score.

A subgroup of patients (n = 37) underwent a symptom limited exercise treadmill testing for clinical indications. A modified Bruce protocol with measurement of peak oxygen consumption (Medgraphics Cardiorespiratory Diagnostic, Ultima Medgraphics, Saint Paul, MN) was used. Respiratory gas analysis was done as breath by breath gas analysis. Continuous EKG monitoring was performed. Stress testing was terminated due to leg fatigue, shortness of breath, or chest pain. The cardiovascular and ventilatory responses to exercise evaluated included changes in heart rate, blood pressure, peak O2 consumption (PVO2), anerobic threshold (AT), and ventilatory efficiency (VE/VCO2) values. Testing was performed after cardiac medications were withheld for four half-lives to avoid their confounding effects on LV function.

### Statistical analysis

Patient characteristics were reported as proportions for categorical variables and as median / interquartile range (IQR) or mean/standardized deviation for continuous variables as applicable. Distribution of continuous variables was tested by The Kolmogorov–Smirnov test. Differences across groups were determined by Chi-square for categorical variables and Student t-test or Mann Whitney U tests for continuous variables as appropriate. Descriptive analyses of MPRI were performed both on a patient and segmental level. Univariate logistic regression analyses were used to determine the characteristics associated with MRPI < 2. Variables having a p-value of < 0.2 in the univariate analysis or considered as clinically important were investigated further by multivariable logistic regression modeling. All the analyses were performed on SPSS version 21 (IBM Corp., Armonk, NY, USA). A p value of < 0.05 was considered statistically significant.

## Results

Baseline patient characteristics are shown in Table 1. The mean age was 53.2 ± 15.4 years and 63 patients (60%) were male. The HCM phenotype was asymmetric hypertrophy in 82, apical in 17, and concentric in 6 patients. Chest pain was reported by 17 (16.2%) patients and dyspnea was reported by 44 (41.9%) patients. In 73 patients who underwent echocardiography, 32 (43.8%) had LVOT obstruction defined as peak gradient ≥ 30 mmHg at rest^19^. None of the patient had prior history of syncope or sudden death. Twenty-two (21%) patients had family history of sudden death. Nineteen patients (18%) had moderate mitral regurgitation (MR) quantified by CMR and three patients had severe MR. The remainder of patients had mild or no MR. One patient had an LVEF of < 50%. Most studies were performed on a 3 T scanner (78%) and the rest were performed on a 1.5 T scanner (22%). The median estimated European Society of Cardiology (ESC) Risk^20^ was 2.2% with IQR of 1.3. Only two patients had an ESC risk ≥ 6%. No adverse events were recorded after Regadenoson infusion. All stress perfusion studies were completed successfully.

**Table 1:** Baseline clinical characteristics.

Characteristics—n (%) or mean (SD)	All patients	No ischemia	Ischemia	P value
	105	58 (55.2)	47 (44.8)	
Age	53.2 (15.4)	54.4 (14.3)	51 (16.6)	0.3
Male gender	63 (60)	37 (63.8)	26 (55.3)	0.4
Caucasian	64 (62.5)	36 (63.2)	29 (61.7)	0.6
ESC risk	2.2 (1.3)	2.3 (1.4)	2.2 (1.3)	0.8
BMI	29.9 (5.9)	30.5 (5.7)	29.1 (6.1)	0.2
Hypertension	57 (54.3)	25 (46.8)	22 (43.1)	0.6
Diabetes	11 (10.5)	9 (15.5)	2 (4.3)	0.1
Current smoker	12 (11.4)	4 (6.9)	8 (17)	0.04
Chest pain	17 (16.2)	10 (17.2)	7 (14.9)	0.6
Dyspnea	44 (41.9)	25 (43.1)	19 (40.4)	0.4
NYHA class III–IV	15 (15.4)	8 (15.1)	7 (15.9)	0.6
Family history of SCD	22 (21)	6 (12.8)	16 (27.6)	0.052
LVOT gradient > 30 mmHg (*)	32 (43.8)	13 (33.3)	19 (63.3)	0.01
LVOT rest gradient (*)	35.6 (38.1)	25.3 (34)	48.6 (39.5)	0.01
Average 16 segment MPRI	3.2 (1.4)	3.9 (1.3)	2.2 (0.7)	0.001

Values are mean ± SD, n (%), or median (interquartile range). The p values are results for the t-test, Mann Whitney U test, or the chi-square test. BMI, body mass index; ESC: European society of cardiology; LVOT, left ventricular outflow tract; MPRI, myocardial perfusion reserve index; SCD, sudden cardiac death. *In patients with echocardiography data.

### Per segment quantitative analysis

**Figure 3:**
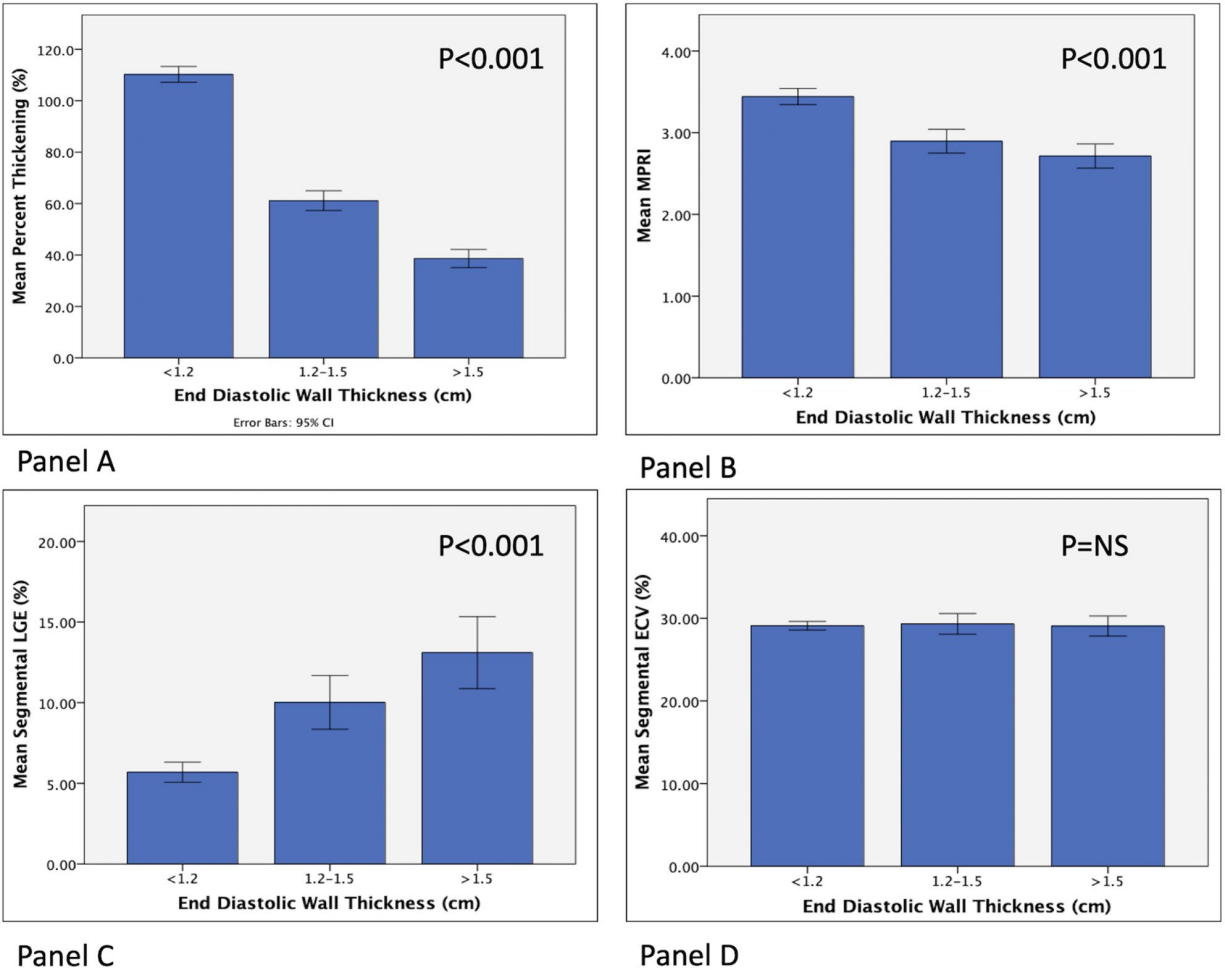
Segmental level analysis according to left ventricular end diastolic wall thickness against percent thickening (panel **A**), MPRI (panel **B**), LGE (panel **C**), and ECV (panel **D**). ECV, extracellular volume fraction; LGE, late Gadolinium enhancement; MPRI, myocardial perfusion reserve index.

Segmental level analysis of all 105 patients included 1,680 segments. The mean end-diastolic wall thickness was 10.6 mm (± 3.9 mm) with 513 (30.5%) of segments having end diastolic wall thickness ≥ 1.2 cm. In comparison to normal thickness segments, segments with end diastolic thickness ≥ 1.2 cm had a higher burden of LGE (4.1% vs 0.5% per segment), reduced MPRI (2.6 ± 1.5 vs 3.1 ± 1.8) and reduced thickening percent (48.9 ± 41.7% vs 105.3 ± 59.5%), (P < 0.0001 for all comparisons). On comparison of segments < 1.2 cm vs ≥ 1.2 cm, segmental ECV was similar (29% vs 28.4%, P = 0.8). Consistent results for all comparisons were noted when comparing segments ≥ 1.5 cm (220 segments, 13.1%) vs. segments < 1.5 cm and when comparing segments ≥ 1.3 cm (400 segments, 23.8%) vs. segments < 1.3 cm (Fig. 3).

**Figure 4:**
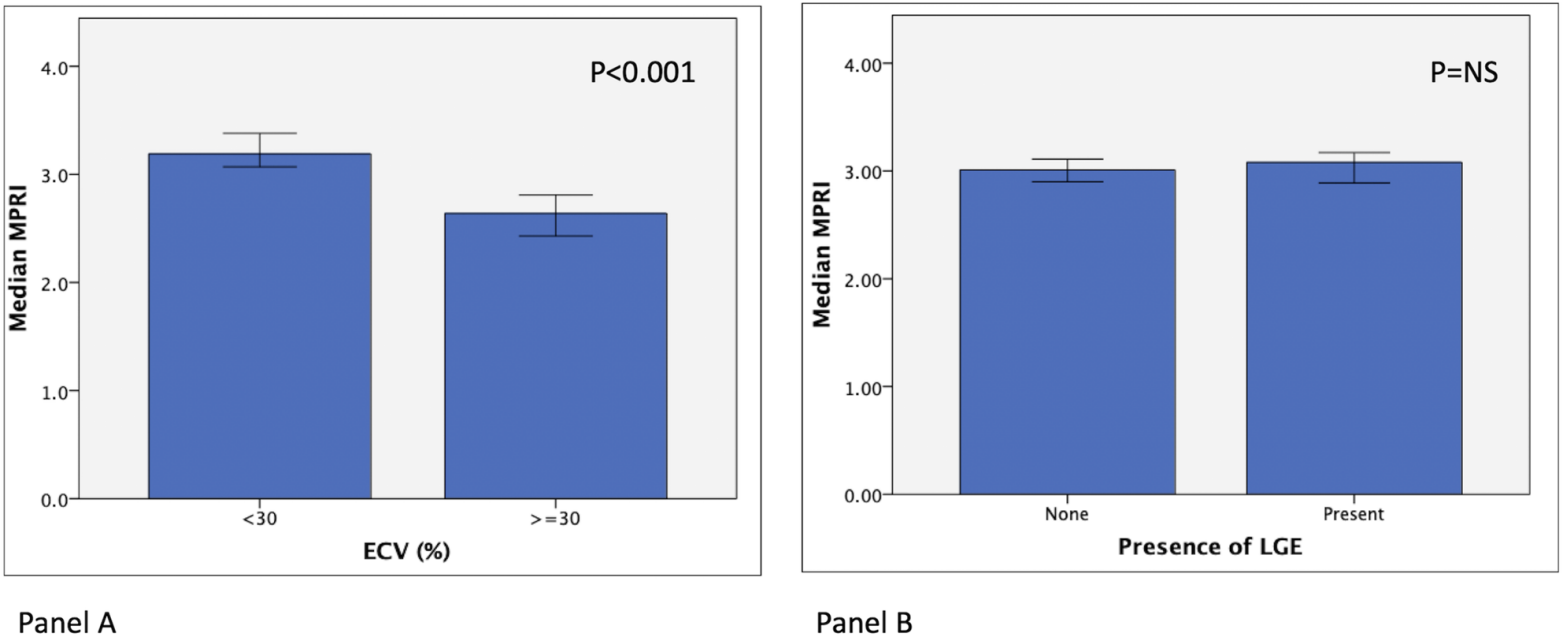
Segmental level analysis of median MPRI according presence of LGE (panel **A**), and ECV (panel **B**). ECV, extracellular volume fraction; LGE, late Gadolinium enhancement; MPRI, myocardial perfusion reserve index.

Segments with high ECV ≥ 30% had a lower MPRI (2.64 ± 1.4 vs 3.1 ± 1.8, P < 0.001) even after excluding segments with any LGE. Segments with LGE had a lower MPRI than segments without LGE (3.12 ± 1.4 vs 3.29 ± 1.6, P = 0.04) (Fig. 4). Univariate predictors of MPRI < 2 included increased end-diastolic wall thickness, decreased segmental thickening, and increased ECV (Table 2). A multivariable logistic regression model showed end-diastolic wall thickness and ECV as independent predictors of MPRI < 2.

**Table 2:** Segmental level univariate predictors of MPRI < 2.

Characteristic	Predictors of MPRI < 2 (segmental level)			
	Univariate OR (95% CI)	P	Multivariate OR (95% CI)	P
End diastolic wall thickness (per cm increase)	2.4 (1.8, 3.3)	0.001	2.9 (1.1, 7.3)	0.02
Thickening percent (per 100% increase)	0.78 (0.62, 0.98)	0.03	1.1 (0.5, 2.4)	0.7
LGE per segment (%)	1.001 (0.99, 1.01)	0.6		
ECV per segment (%)	1.04 (1.004, 1.097)	0.03	1.05 (1.007, 1.1)	0.02

ECV, extracellular volume fraction; LGE, late Gadolinium enhancement; MPRI, myocardial perfusion reserve index.

### Global ventricular measures

Forty-seven patients (44.8%) had at least one segment with MPRI < 2 and were considered the group with ischemia. The mean number of segments with MPRI < 2 was 6.7 ± 5.3 segments. Global ventricular measures are shown in Table 3. Left and right ventricular indexed volumes and ejection fraction measures were similar between the two groups (P ≥ 0.2 for all comparisons). The distribution of apical hypertrophy, asymmetric hypertrophy, and concentric hypertrophy was similar among the ischemic and non-ischemic groups (P = 0.3). LGE was present in 90 (85.7%) of patients. The mean number of segments with LGE was 4.1 ± 3.7 segments.

**Table 3:** Global ventricular measures in patients with and without ischemia.

Characteristics—n (%) or mean (SD)	All patients	No ischemia	Ischemia	P value
	105	58 (55.2)	47 (44.8)	
Apical hypertrophy	17 (16.2)	9 (15.5)	8 (17)	0.3
LVEDV (indexed)	32.5 (8)	32.4 (9)	32.5 (7.8)	0.9
LVESV (indexed)	14.7 (9.6)	15.3 (9.4)	13.9 (10.5)	0.2
LV mass (indexed)	105 (34.5)	107.4 (36.2)	109.1 (32.7)	0.8
LVEF	75.8 (7.4)	75.2 (7.1)	76.6 (7.7)	0.3
RVEDV (indexed)	63.6 (22.4)	64.7 (25.8)	63.6 (19.4)	0.3
RVESV (indexed)	23.1 (13.6)	24.6 (14.7)	29.9 (12.2)	0.4
RVEF	63. (9.5)	64 (10.1)	62.1 (9.4)	0.8
ECV (%)	0.29 (0.041)	0.28 (0.037)	0.30 (0.044)	0.04
Total LGE burden (% of LV mass)	4 (8.5)	4 (8.3)	5 (10)	0.4

The p values are results for the t-test, Mann Whitney U test, or the chi-square test.

ECV, extracellular ejection fraction; LGE, late Gadolinium enhancement; LVEDV, left ventricular end diastolic volume; LVESV, left ventricular end systolic volume; LVEF, left ventricular ejection fraction; RVEDV, right ventricular end diastolic volume; RVESV, right ventricular end systolic volume; RVEF, right ventricular ejection fraction.

In comparison to patients without ischemia, patients with ischemia were more likely to have dynamic LVOT obstruction (63.3% vs 33.3%, P = 0.01), and have a higher resting LVOT gradient (48.6 ± 39.5 vs 25.3 ± 34 mmHg, P = 0.01). There were more smokers in the ischemia group (17% vs 6.9%, P = 0.04). There was a trend in the ischemia group to have family history of sudden death (P = 0.052). No significant differences in NYHA class were found. The total LGE burden was similar between the two groups (P = 0.4) but ECV was higher in the ischemic group (30% vs 28%, P = 0.04).

### Echocardiographic parameters and exercise capacity

**Figure 5:**
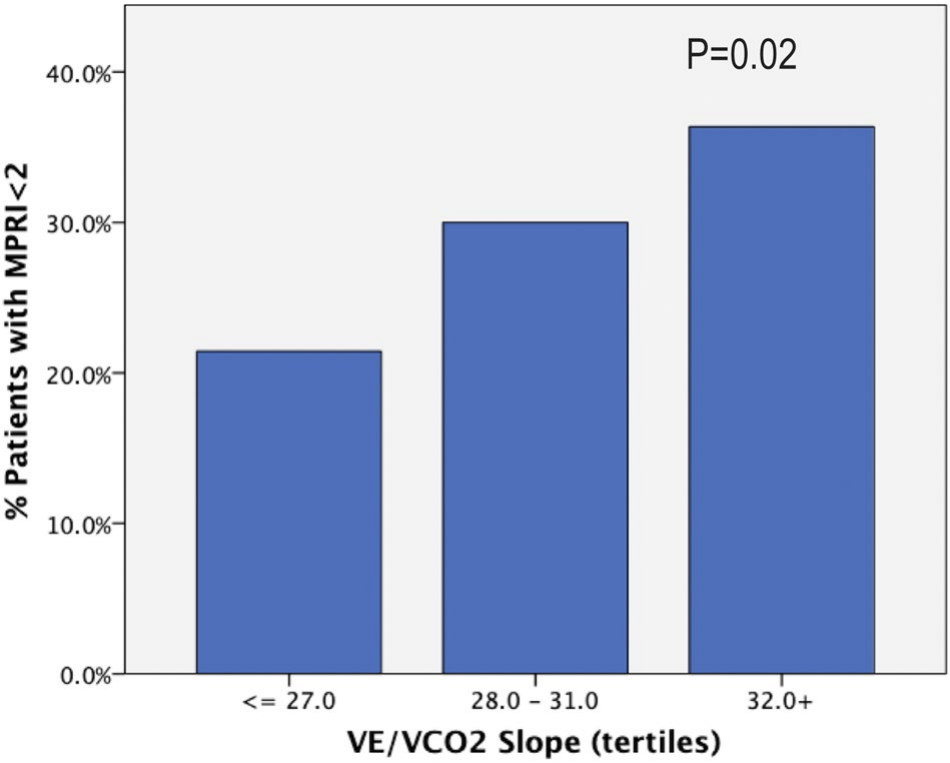
Patient level analysis to examine the association of increased burden of ischemia (assessed by number of segments with MPRI < 2) and worsened ventilatory efficiency (higher is worse). P value is for multivariable linear regression controlling for baseline heart rate. MPRI, myocardial perfusion reserve index; VE/VCO2, ratio of minute ventilation to CO2 production or ventilatory efficiency.

In patients who underwent exercise testing, no significant differences were noted in exercise duration in the ischemic vs non-ischemic group (11:15 ± 5:30 vs 11:26 ± 4:38 min:sec, P = 0.9), peak VO2 consumption (20.2 ± 7.1 vs 23.3 ± 9.8 ml/kg/min, P = 0.34) vs or anaerobic threshold (10.9 ± 3.6 vs 12.8 ± 4.2 ml/kg/min, P = 0.20). But VE/VCO2 was higher in the group with ischemia (P = 0.05), and increasing ischemia burden (number of segments with MPRI < 2) was associated with worsened ventilatory efficiency VE/VCO2 (P < 0.001) (Fig. 5). No significant associations were noted between reduced MPRI and diastolic function parameters or echocardiographic estimated left ventricular filling pressure (data not shown).

In a patient level multivariate logistic regression model correcting for age, gender, anteroseptal wall thickness, indexed LV mass, VE/VCO2 and ECV > 30%, only LVOT obstruction remained a significant predictor of reduced MPRI (Table 4).

**Table 4:** Patient level multivariable regression model to predict extent of ischemia (number of segments with MPRI < 2).

Predictors of MPRI < 2 (patient level)		
Characteristic	Multivariate OR (95% CI)	P
Age (years)	0.93 (0.84, 1.0)	0.14
Female gender	0.108 (0.001, 11.9)	0.3
VE/VCO2 slope	1.22 (0.85, 1.75)	0.2
LV mass index (g/m^2^)	0.92 (0.84, 1.02)	0.1
Anteroseptal wall thickness (mm)	0.60 (0.01, 27.1)	0.7
ECV > 30%	2.9 (0.06, 128.8)	0.5
LVOT gradient (mmHg)	1.05 (1.005, 1.1)	0.03

VE/VCO2, ventilatory efficiency; ECV, extracellular volume fraction; LVOT, left ventricular outflow tract.

## Discussion

We found that reduced myocardial perfusion reserve by vasodilator stress CMR was associated with myocardial segmental function and interstitial fibrosis, measured by ECV, in low-intermediate risk HCM patients. Reduced MRPI correlated with increased wall thickness, reduced segmental wall thickening, increased ECV, even in myocardial segments free of LGE. Patients with ischemia had greater prevalence of dynamic LVOT obstruction and a higher resting LVOT gradient compared to patients without ischemia. We also assessed the impact of myocardial ischemia on exercise capacity and found a higher burden of ischemia to be associated with worsened exercise capacity assessed by ventilatory efficiency, but not peak VO2, exercise duration, or anaerobic threshold.

Patients with HCM have long been known to have intramural small vessel disease and myocardial fibrosis by histopathological studies. Maron et al.^5^. described thickening of the vessel walls and a decrease in luminal size. The wall thickening was related to proliferation of medial or intimal components, or both, especially smooth muscle cells and collagen. Small vessel disease was also significantly more common in segments having considerable myocardial fibrosis, which could account for the association of increased ECV in segments with decreased MRPI reported in our study. However, in contrast to our study, the severity of small vessel disease was not different in those with obstructive HCM vs non-obstructive HCM. A potential explanation could be the additive effect of altered coronary hemodynamics in obstructive HCM^7^, which could only be studied in vivo and could worsen myocardial ischemia in segments with existing small vessel disease. Factor et al.^21^ also described areas of interstitial, perivascular and replacement fibrosis in specimens of HCM patients, with markedly increased collagen and strikingly increased and disorganized matrix connective tissue.

The direct visualization and quantitation of replacement fibrosis by LGE techniques has become essential in risk-stratifying HCM patients. Vasodilator stress CMR can also detect and quantify perfusion abnormalities in HCM, which were recently linked to LGE progression^13^. Our findings are consistent with prior CMR studies in showing that myocardial ischemia, reduced thickening, and LGE is more prevalent in hypertrophied segments^10,22^. In addition, we show that ischemia is associated with LVOT obstruction and associated with increased interstitial fibrosis. Despite segments with LGE having a statistically significant reduction in MPRI compared to segments without LGE in our study, the overall burden of LGE was similar between the ischemia and no ischemia groups, in contrast to other studies^23^. Possible explanations for this difference include a smaller sample size in other studies, residual enhancement in areas of LGE when performing rest imaging (due to contrast given for the stress perfusion imaging) which may affect MRPI calculation, and importantly, that pathophysiologic mechanisms behind ischemia and LGE are not completely interlinked. Based on the association of CMR detected ischemia with interstitial fibrosis and reduced segmental function, we hypothesize that assessment of ischemia could aid in the detection of early features of decompensation towards heart failure in HCM patients. However, further longitudinal studies are needed to further explore this. Considering the association between ischemia burden and LVOT obstruction, intensification of therapy to alleviate LVOT obstruction may have an effect on reducing ischemic burden, although whether this impacts clinical outcomes requires further study. The complex and heterogenous etiologies of ischemia in HCM certainly warrant further investigation.

In our study, myocardial ischemia was associated with decreased ventilatory efficiency on CPET, which is known to be a poor prognostic marker both in HF and in HCM^24^. However, we did not find significant differences in exercise duration, peak VO2 or the anaerobic threshold between the ischemic and non-ischemic groups, although the latter two were slightly lower in the group with ischemia. This may be due to the small number of patients with exercise data and confirmation is needed in larger studies.

Our study has several limitations: this is a cohort from a tertiary referral center and selection bias limits the generalizability of the findings. However, the low-intermediate risk population is likely the group where stress CMR can influence management and frequency of monitoring. We used semi-quantitative measures of ischemia rather than absolute measures of myocardial blood flow, however, semi-quantitative measures were found to be more reproducible than quantitative methods and could be performed with commercially available software^25^. Semi-quantitative measures were also validated against microspheres albeit not in HCM^26^. We did not assess differences in MRPI in the subendocardium vs subepicardium in our study. However, studies by Tyan et al.^22^, Petersen et al.^27^, and others have established myocardial ischemia to predominate in the subendocardial zones. Data on exercise capacity was limited to a small subgroup which limited analysis; and Holter monitoring, genotyping, and clinical outcomes were not described. As this is a relatively low risk population, we observed only 3 deaths on follow up. Only a minority of patients enrolled underwent coronary angiography to rule out epicardial coronary disease. However, patients with LGE or ischemia in a coronary distribution were excluded; and clinical history and available diagnostic testing results were thoroughly evaluated to minimize confounding. No control population was used in this study because our primary aim was to compare HCM patients with and without ischemia and multiples studies have already established the differences between HCM patients and healthy controls.

## Conclusions

We found that reduced myocardial perfusion reserve measured by vasodilator stress CMR was associated with myocardial segmental function and myocardial interstitial fibrosis, as assessed by ECV, in low-intermediate risk HCM patients. While segments with LGE had slightly lower MPRI, quantitative total ischemia burden was not associated with replacement fibrosis as total LGE burden. Reduced MRPI correlated with increased wall thickness, reduced segmental wall thickening, increased ECV, even in myocardial segments free of LGE. Patients with ischemia have greater prevalence of dynamic LVOT obstruction. In the subset of patients who underwent cardiopulmonary exercise testing, presence of ischemia was associated with worsened ventilatory efficiency but no significant difference in exercise duration or peak VO2.
